# CRMP1 Inhibits Proliferation of Medulloblastoma and Is Regulated by HMGA1

**DOI:** 10.1371/journal.pone.0127910

**Published:** 2015-05-26

**Authors:** Kay Ka-Wai Li, Yan Qi, Tian Xia, Yu Yao, Liangfu Zhou, Kin-Mang Lau, Ho-Keung Ng

**Affiliations:** 1 Department of Anatomical and Cellular Pathology, The Chinese University of Hong Kong, Hong Kong, Prince of Wales Hospital, 30–32 Ngan Shing Street, Shatin, Hong Kong, China; 2 Shenzhen Research Institute, The Chinese University of Hong Kong, No.10, 2nd Yuexing Road, Nanshan District, Shenzhen, China; 3 Department of Neurosurgery, Huashan Hospital, Fudan University, Wulumuqi Zhong Road 12, Shanghai, China; University of Navarra, SPAIN

## Abstract

Many facets of the tumor biology of medulloblastoma (MB) have not been fully elucidated. Collapsin response mediator protein 1 (CRMP1) is a member of cytoplasmic family of proteins that regulate the development of central nervous system. Recent studies demonstrated that CRMP1 could function as an invasion suppressor. We reported previously that high mobility group AT-hook 1 (HMGA1) contributed to development of MB and regulated its growth and migration/invasion. Transcriptional profiling and quantitative RT-PCR revealed increased expression of CRMP1 in HMGA1-depleted cells, suggesting that CRMP1 may be a downstream target of HMGA1 in MB. In this study, we showed HMGA1 can bind CRMP1 promoter by chromatin immunoprecipitation (ChIP) assay. Luciferase assay demonstrated a marked enhancement of CRMP1 transcription activity in HMGA1-depleted cells. Furthermore, quantitative RT-PCR revealed a negative correlation between HMGA1 and CRMP1 in 32 MB samples. To investigate the biological roles of CRMP1 in MB pathogenesis, we established MB clones stably expressing CRMP1. Functional analysis revealed that expression of CRMP1 significantly inhibited proliferation, migration, invasion and formation of filopodia and intense stress fiber of MB cells. Our data suggest that HMGA1 regulates CRMP1 expression and CRMP1 is implicated in MB pathogenesis.

## Introduction

Medulloblastoma (MB) is the most common malignant central nervous system neoplasm of children. The World Health organization (WHO) classification of MB recognizes five histological variants, namely, classic MB, desmoplastic/nodular MB, MB with extensive nodularity (MBEN), large cell (LC) MB, and anaplastic (A) MB [1]. For a quite a long time, risk stratification of MB has been based on age, metastatic stage at diagnosis and extent of surgical resection. With current multimodal treatment comprised of surgical resection, radiotherapy and chemotherapy, outcome of average risk patient is satisfactory, achieving an around 90% in five year overall survival [2]. However, outcome remains poor in high-risk patients despite intensive treatment regime [3]. Yet, the cost of therapy is high; patients suffer from long-term neurocognitive and neuroendocrine defects [4–5]. Advancement in high-throughput genomic technology enables the identification of molecular subgroups in MB. What was once thought of as a single disease can now be categorized into four principal subgroups, namely, WNT, SHH, Group 3, and Group 4 [6–10]. Each of these subgroup is characterized by distinct transcription signature, chromosomal aberration, demographic features, and clinical outcomes.

In view of the significant mortality and morbidity associated with treatment, there is an urgent need to unravel as much as possible the molecular pathogenesis of MB so that rational classification and treatment regimen can be instituted. Collapsin response mediator protein 1 (CRMP1) is a brain specific phosphoprotein and a member of the CRMP family of cytosolic proteins. It is differentially expressed in the developing and adult central nervous system [11–12]. CRMP1 is highly expressed in the developing cerebellum, olfactory bulbs, hypothalamus and retina [13–14]. However, it is expressed at low level in the retina and cerebellum of adults [13]. The gene is also critical in neuronal development and maturation [15]. It regulates neuronal network formation, migration, extension and differentiation, and affects growth cone collapse in migratory neurons [16–21].

Cumulative evidence indicates that CRMP1 also contributes to tumor pathogenesis. Dysregulation of CRMP1 has been reported in brain, lung, and prolactin pituitary tumors [22–24]. In prolactin-secreting pituitary adenoma, CRMP1 was associated with tumor progression [25]. Downregulation of CRMP1 was significantly associated with advanced disease, metastasis, and shorter survival in non-small cell lung cancer (NSCLC), suggesting that CRMP1 may act as a novel invasion suppressor gene [26–27]. Functional studies demonstrated that depletion of CRMP1 enhanced tumor invasion, whereas increased expression had an opposite effect in glioblastoma [23]. In NSCLC, expression of CRMP1 led to reduction of invasive activity, change in morphology and decrease in filopodia formation [26].

We previously reported that an architectural transcription factor HMGA1 is upregulated in MB and plays a role in cell proliferation, migration, and invasion [28]. Global gene expression analysis indicated that CRMP1 was upregulated in HMGA1-silenced MB cells. The molecular mechanisms by which HMGA1 mediates CRMP1 are not understood. In this study, we showed that HMGA1 negatively regulates CRMP1 through direct binding at the distal region of CRMP1 promoter. We detected an inverse correlation between transcript abundance of HMGA1 and CRMP1 in MB samples. Finally, we studied the functional importance of CRMP1 in MB biology by using stably expressing CRMP1 clones derived from three MB cell lines. And, we demonstrated that cell proliferation, migration, invasion, and formation of filopodia and intense stress fibers were inhibited in CRMP1 expressing cells.

## Materials and Methods

### Tumor samples

A cohort of 32 primary MBs were recruited from Huashan Hospital, Shanghai. Tumor tissues were collected at the time of surgical resection and frozen in RNALater solution (Ambion, Inc., Austin, TX, USA) at -80°C until further use. All tumors were reviewed by a pathologist and classified according to current WHO criteria [1]. There were 25 classic MB, 4 desmoplastic MB, and 3 anaplastic MB. The cohort comprised 23 children and 9 adult patients. The median age of children group was 9 years old (range, 5–16) and of adult group was 24 years old (range, 19–50). The clinicopathological information of the patients is summarized in S1 Table.

### RNA extraction

RNA was extracted using RNeasy Plus Mini Kit (Qiagen, Valencia, CA, USA) according to the manufacturer's protocol.

### Quantitative RT-PCR

Expression of HMGA1 and CRMP1 was assessed by quantitative RT-PCR. cDNA was synthesized from 1μg RNA using MultiScribe reverse transcriptase and random hexamers as described by manufacturer (Applied Biosystems, Foster City, CA, USA). Amplification was carried out in a reaction mixture contained cDNA, 1xTaqMan Universal PCR master mix, and CRMP1 TaqMan Gene Expression Assays (Hs00609717_m1), HMGA1 (Hs00852949_g1), or GAPDH (Hs99999905_m1) (Applied Biosystems), and run in triplicate with an ABI 7900HT Fast Real-time PCR system (Applied Biosystems). Relative expression level for target genes was normalized by the Ct value of GAPDH (internal control) using a 2^-ΔΔCt^ relative quantification method.

### Cell culture

DAOY, D283, and D341 were obtained from American Type Culture Collection (Manassas, VA, USA). ONS-76 was purchased from Japanese Cancer Research Resources Bank. UW228-1 was generous a gift from Dr. John Silber (University of Washington, Seattle, WA, USA). D384, D425 and D458 were kind gifts from Dr. Darrell Bigner (Department of Pathology, Duke University, Durham, NC, USA). UW228-1 was derived from a patient with MB in posterior fossa [29]. D384 was derived from a 18-months-old boy with MB in posterior fossa [30]. D425 and D458 were isolated from the same 6-years-old boy with cerebellar MB at different times [30]. UW228-1, D384, D425, and D458 were immunopositive for neurofilament proteins (NFP) and synaptophysin, but were immunonegative for glial fibrillary acidic protein (GFAP) [29–30]. Cell lines were maintained in recommended media supplemented with 10% fetal bovine serum, and housed at 37°C in a 95% air / 5% CO_2_ incubator.

### CRMP1 promoter-luciferase constructs

Three luciferase reporter constructs were generated to study the regulation of HMGA1 on CRMP1 promoter. These included (1) pCRMP1-full which contained nts -2932 to -279 of the CRMP1 gene (+1 denoted to the the transcription start site); (2) pCRMP1-distal that contained nts -2932 to -1734 of the CRMP1 gene; and, (3) pCRMP1-proximal that contained nts -1733 to -279 of the CRMP1 gene. Genomic DNA fragments of these regions were obtained by PCR amplification using AmpliTaq Gold DNA Polymerase (Applied Biosystems, Branchburg, New Jersey, USA) with primer sequences listed in S2 Table. PCR products of pCRMP1-full, pCRMP1-distal, and pCRMP1-proximal were digested with KpnI/HindIII, KpnI/NheI, and NheI/HindIII respectively. The resultant products were cloned into corresponding restriction enzyme-digested pGL3-basic vector (Promega Co., Madison, WI, USA). Sanger sequencing was performed on an ABI 3130xl Genetic Analyzer (Applied Biosystems) to confirm inserted DNA sequence. Restriction enzyme digestion was conducted to verify plasmid size.

### Promoter activity assay

A day prior of transfection, 4.0–6.0x10^4^ DAOY and ONS-76 cells were seeded on 24-well plate at a density that reached 90% confluence at transfection. Cells were then cotransfected with 0.5μg of pCRMP1-full, pCRMP1-distal, or pCRMP1-proximal plasmids and 20pmol of siRNA targeting HMGA1 (Invitrogen, Carlsbad, CA, USA) or Silencer Negative Control #1 siRNA (Invitrogen) using Lipofectamine 2000 (Invitrogen). pRL-CMV renilla plasmid (Promega) was included as internal control to normalize transfection efficiency. After 48h incubation, the firefly luciferase activity was measured with Dual Luciferase Reporter Assay System (Promega). Relative luciferase unit (RLU) was calculated as the ratio of firefly to renilla luciferase activities. The experiments were repeated three times and three replicate measurements were taken in each test.

### Chromatin immunoprecipitation (CHIP) assay

HMGA1-bound chromatins were examined by EZ ChIP Chromatin Immunoprecipitation kit (Millipore, Billerica, MA, USA) according to manufacturer’s recommended protocol. In brief, DAOY cells were fixed with 1% formaldehyde to crosslink proteins to DNA. Glycine was added to stop crosslinking, and cells were washed with ice-cold 1xPBS containing Protease Inhibitor Cocktail II. Cell pellets were then resuspended in SDS Lysis Buffer containing 1xProteinase Inhibitor Cocktail II and sonicated on ice. The sheared cross-linked chromatin was precleared with Protein G Agarose beads and immunoprecipitated with anti-HMGA1 antibody (abcam, Cambridge, UK) or mouse IgG as negative antibody control. The protein-DNA complexes were eluted with freshly prepared elution buffer (1% SDS and 100mM NaHCO_3_). Eluted samples were incubated with 0.2M NaCl at 65°C overnight, treated with RNase A at 37°C and Proteinase K at 45°C for 1h each, purified using spin columns, and then subjected to PCR amplification by AmpliTaq Gold (Applied Biosystems). The primers for distal CRMP1 promoter were 5’- AAGGCGCTTTGCTCTCTTG -3’ and 5’- GAGTTCCACAGTCGCGAAG -3’. The primers for proximal CRMP1 promoter were 5’- CCCGGGGTACATCATTTTAC -3’ and 5’- CCAAGTTCCCAGGCAGAATA -3’.

### Generation of CRMP1 expressing vector

The 1887 bp cDNA fragment of CRMP1 (NM_001313.4) was amplified using forward primer 5’- C*AAGCTT*CCTCCGTCCGTGTCTCTATC -3’ and reverse primer 5’- C*CTCGAG*TCCCAGAATCCTTCAGGCTA -3’ with KAPA 2G Robust DNA Polymerase (Kapa Biosystems, Wilmington, MA, USA). The italic letters represent the restriction enzyme sites HindIII and XhoI. The PCR product was first cloned into pCR2.1 TOPO (Invitrogen), and then subcloned into pcDNA3.1 (+) vector. The resultant plasmid was named pcDNA3.1-CRMP1. The sequence was confirmed by DNA sequencing. The plasmid size was validated by restriction enzyme cut.

### Establishment of stable clones

6.0x10^4^ DAOY, 1.0x10^5^ ONS-76, and 6.0x10^4^ UW228-1 cells were transfected with pcDNA3.1-CRMP1 or pcDNA3.1 control plasmids using Lipofectamine 2000 (Invitrogen). The next day, cells were then incubated with G418 at a final concentration of 400μg/ml for selection of stably transfected clones. The antibiotic containing medium was changed every 2–3 days. Viable clones were visible 2–4 weeks after transfection. Clones stably expressing CRMP1 were further examined by quantitative RT-PCR and western blot analysis. Two vector-transfected clones (called control) and two CRMP1 stable clones (called CRMP1) of each cell line were used in this study.

### Western blot analysis

Established stable clones were lysed in RIPA protein lysis buffer [50mM Tris-HCl (pH 7.6), 150mM NaCl, 1% NP-40, 4 mM EDTA, 1% sodium deoxycholate, 0.1% SDS, 1xComplete Protease Inhibitors Cocktail]. Protein concentrations were measured using Bio-Rad Protein Assay Kit (Bio-rad, Hercules, CA, USA). A total of 10μg of protein lysates were loaded on 10% SDS-PAGE gel and transferred onto polyvinylidene difluoride (PVDF) membrane (GE Healthcare, Buckinghamshire, UK). The membrane was incubated with anti-CRMP1 (1:2000; Abcam) or anti-β-ACTIN (1:10000; Sigma) and then with HRP-conjugated secondary antibodies. Immobilon Western Chemiluminescent HRP substrate western blotting reagent (Millipore) was employed to visualize the protein bands.

### Cell proliferation assay

Stably transfected cells were examined for cell proliferation using 3-(4,5-dimethylthiazol-2-yl)-2,5-diphenyltetrazolium bromide (MTT)-based assay. Briefly, a total of 1.5x10^3^ DAOY, 0.8x10^3^ ONS-76, and 1.2x10^3^ UW228-1 cells were seeded onto 96-wells culture plates in a total volume of 100μl. After 3–4 days cell culture, medium was replaced, and cell proliferation was assessed by Vybrant MTT cell proliferation assay kit (Invitrogen). A total of 10μl MTT solution was added into each well and incubated at 37°C for 3h. The solution was then removed and replaced with 100μl DMSO (MP Biomedicals, Santa Ana, CA, USA) for 30min at 37°C. Absorbance was measured at 560nm by a Glomax Multi-Detection system (Promega). In an experiment, each sample had four replicas and the experiment was repeated at least three times.

### Migration assay

Migration assay was examined by uncoated transwell inserts with 8μm pore size (BD Bioscience, Bedford, MA, USA). Stably transfected clones were harvested, counted, resuspended in serum-free culture media, and added to the upper compartment of inert at a density of 1.5–2.5x10^4^ cells. Culture media containing 10% FBS as chemoattractant was filled in the lower compartment of insert. Cells were then incubated at 37°C in a 95% air / 5% CO_2_ incubator for 22h to allow cell migration across the membrane. The non-migrated cells on the inner side of the upper compartment were removed by moistened cotton swabs. The migrated cells were fixed by 100% methanol and stained with 2% crystal violet. The number of migrated cells was counted under a light microscope at a magnification of x200. The experiment was repeated four times independently.

### Cell invasion assay

Cell invasion was determined by BD BioCoat Matrigel Invasion Chamber (BD Bioscience). Stably transfected clones were seeded on the upper compartment of rehydrated Matrigel-coated invasion chamber at a density of 2.5–4.0x10^4^ cells. Cells were incubated for 22h, and non-invading cells were removed. Invading cells passed to the lower compartment of chamber were fixed by 100% methanol and stained with 2% crystal violet. The number of invaded cells was counted under light microscope at a magnification of x200. The experiment was repeated four times independently.

### Filamentous-actin (F-actin) staining

The formation of filopodia/intense stress fibers was assessed by F-actin staining according to the previous study [28]. In brief, stably transfected clones were plated onto poly-D-lysine-coated coverslips and allowed to grow in culture media for 24h. The next day, cells were fixed in 3.7% formaldehyde for 10-30min, washed with PBS for 3 times, and permeabilized with 0.1% Triton X-100 in PBS (PBS-T) for 10min at room temperature. To visualize F-actin, cells were then incubated with Tetramethyl Rhodamine Isothiocyanate (TRITC)-conjugated phalloidin (1:10,000; Sigma Chemical Co., St. Louis, MO, USA) for 30min. Images were captured and collected by a laser confocal microscope LSM5 PASCAL (Carl Zeiss, Oberkochen, Germany).

### Statistical analysis

For continuous variable, data are shown as mean and standard deviation. Data were analyzed by Student’s t test for comparison between two groups, and one-way ANOVA followed by Bonferroni test for comparison of multiple groups. Spearman rank correlation analysis was used to analyze the association between HMGA1 and CRMP1. All p values quoted were two-sided and significant was considered if p<0.05.

## Results

### HMGA1 was negatively associated with CRMP1 in MB

**Fig 1:**
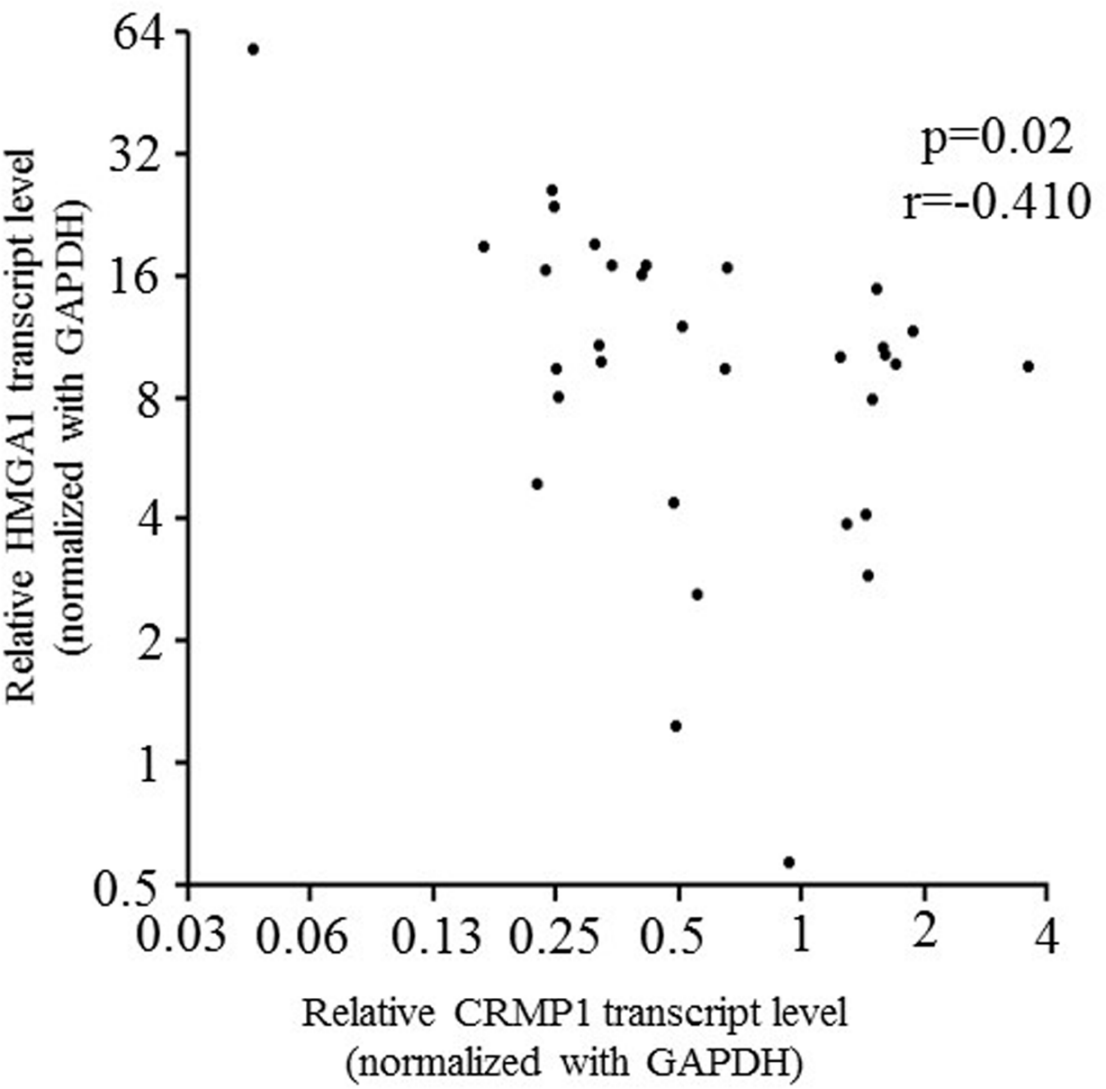
An inverse correlation between HMGA1 and CRMP1 transcript level in 32 MB tumors. Expression of HMGA1 and CRMP1 were examined by quantitative RT-PCR. The Spearman's correlation coefficient between HMGA1 and CRMP1 was -0.410 (p = 0.02).GAPDH was served as internal control.

**Fig 2:**
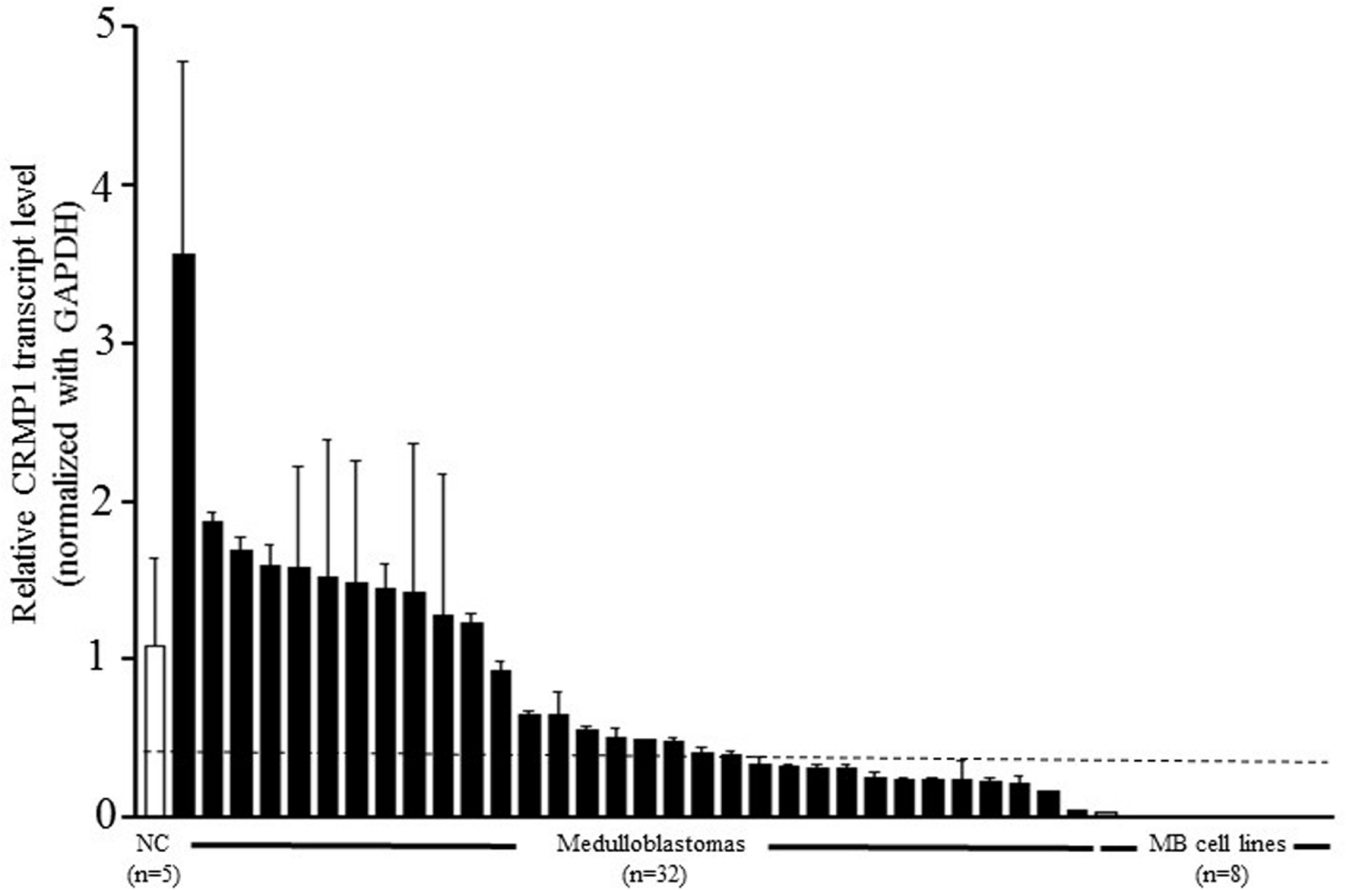
Reduced transcript level of CRMP1 in MB tumors and cell lines. Expression of CRMP1 was measured by quantitative RT-PCR in 32 primary MBs (black bar), 8 cell lines (grey bar) and 5 normal cerebella (white bar). Compared to the mean of 5 normal cerebella, CRMP1 was significantly lower in 12/32 (37.5%) of MB and 8/8 (100%) of MB cell lines. The samples below the dot line were detected with reduced CRMP1 expression. The bar represents the mean of three measurements and the error bars indicates the standard deviation (SD).

As reported previously, we conducted gene expression profiling of HMGA1-depleted MB cells to identify potential downstream target genes [28]. By quantitative RT-PCR, we confirmed that CRMP1 was upregulated in HMGA1-depleted DAOY cells [28]. We speculated that CRMP1 is negatively regulated by HMGA1 in MB. To address this hypothesis, we first conducted quantitative RT-PCR to examine transcript levels of HMGA1 and CRMP1 in a cohort of 32 MB samples and 5 normal cerebella. As depicted in Fig 1, expression of HMGA1 was negatively correlated with that of CRMP1 (p = 0.02; r = -0.410), supporting our hypothesis that HMGA1 negatively regulates CRMP1. Furthermore, 12/32 (37.5%) of MB and 8/8 (100%) of MB cell lines displayed prominently decreased CRMP1 expression when compared to the average of normal cerebella (Fig 2). We did not observe association between CRMP1 mRNA level and histology, molecular subgroups, progression free survival, or overall survival.

**Fig 3:**
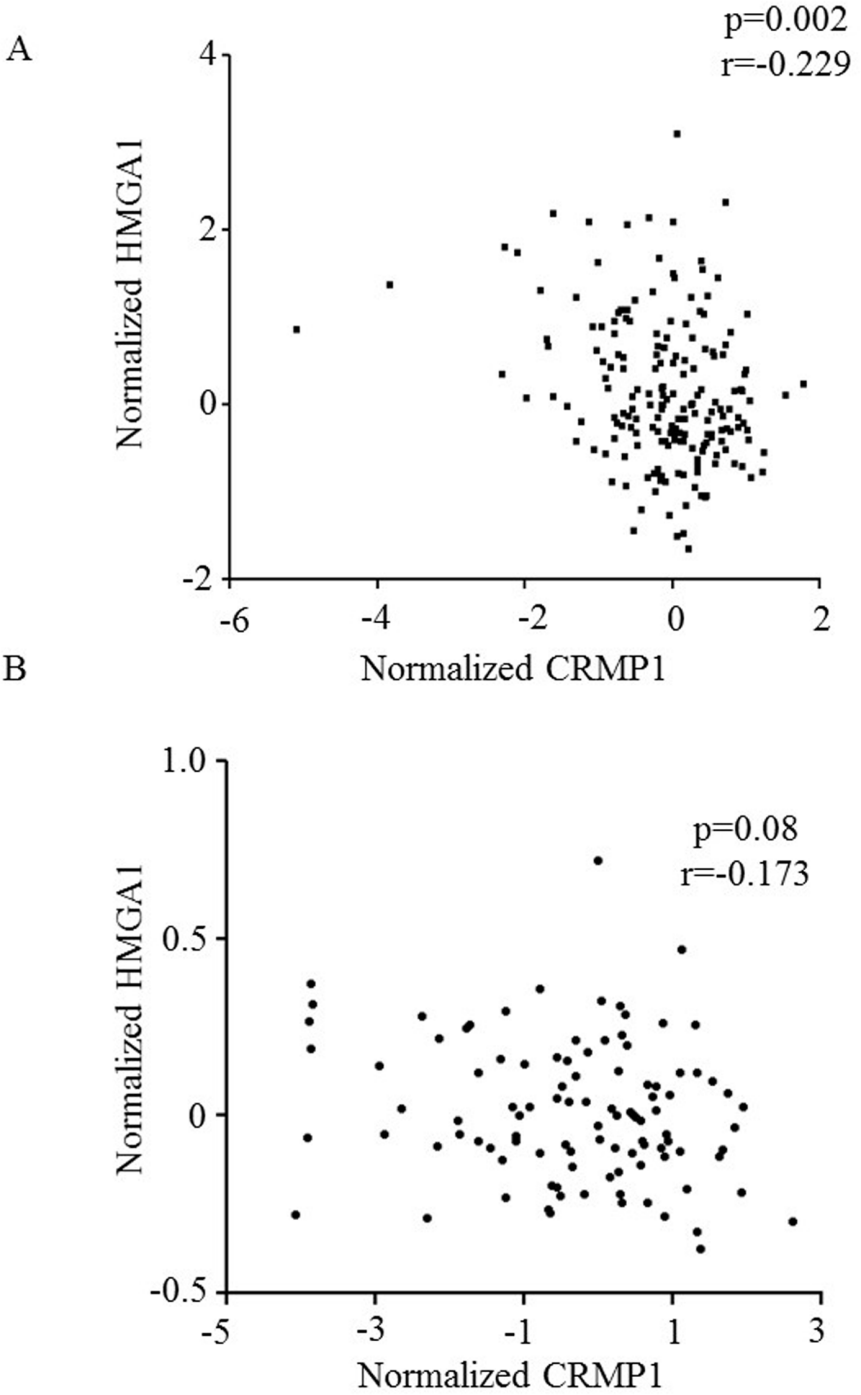
An inverse correlation between HMGA1 and CRMP1 expression as revealed by expression profiling studies. Expression data were retrieval from (A) Cho et al. study which comprised of 189 MB and (B) Northcott et al. study which comprised of 103 MB. Correlation coefficients were determined with Pearson's correlation analysis.

To further provide evidence to show the regulatory role of HMGA1, we accessed to a global gene expression array dataset of 189 MB at http://www.broadinstitute.org/pubs/medulloblastoma/cho, which was generated in Cho et al. study [6]. We extracted HMGA1 and CRMP1 expression for correlation analysis and found an inverse correlation between HMGA1 and CRMP1 (Fig 3A; p = 0.002; r = -0.229). We further attempted to validate our finding in another transcriptome dataset GSE21140 comprised of 103 MB samples from NCBI database [9]. We found a trend towards a negative association between HMGA1 and CRMP1 (Fig 3B; p = 0.08; r = -0.173). We reasoned that differences in expression platform and probe design contributed to the approach significance finding. Interestingly, both transcriptome datasets demonstrated a significant reduced CRMP1 expression in MB with Group 3 molecular subgroup (S1 Fig; p<0.01; ANOVA). CRMP1 expression was not associated with clinical outcomes in both cohorts of expression array samples. Taken together, we demonstrated an inverse relationship between the expression of HMGA1 and CRMP1.

### Depletion of HMGA1 altered CRMP1 promoter activity

**Fig 4:**
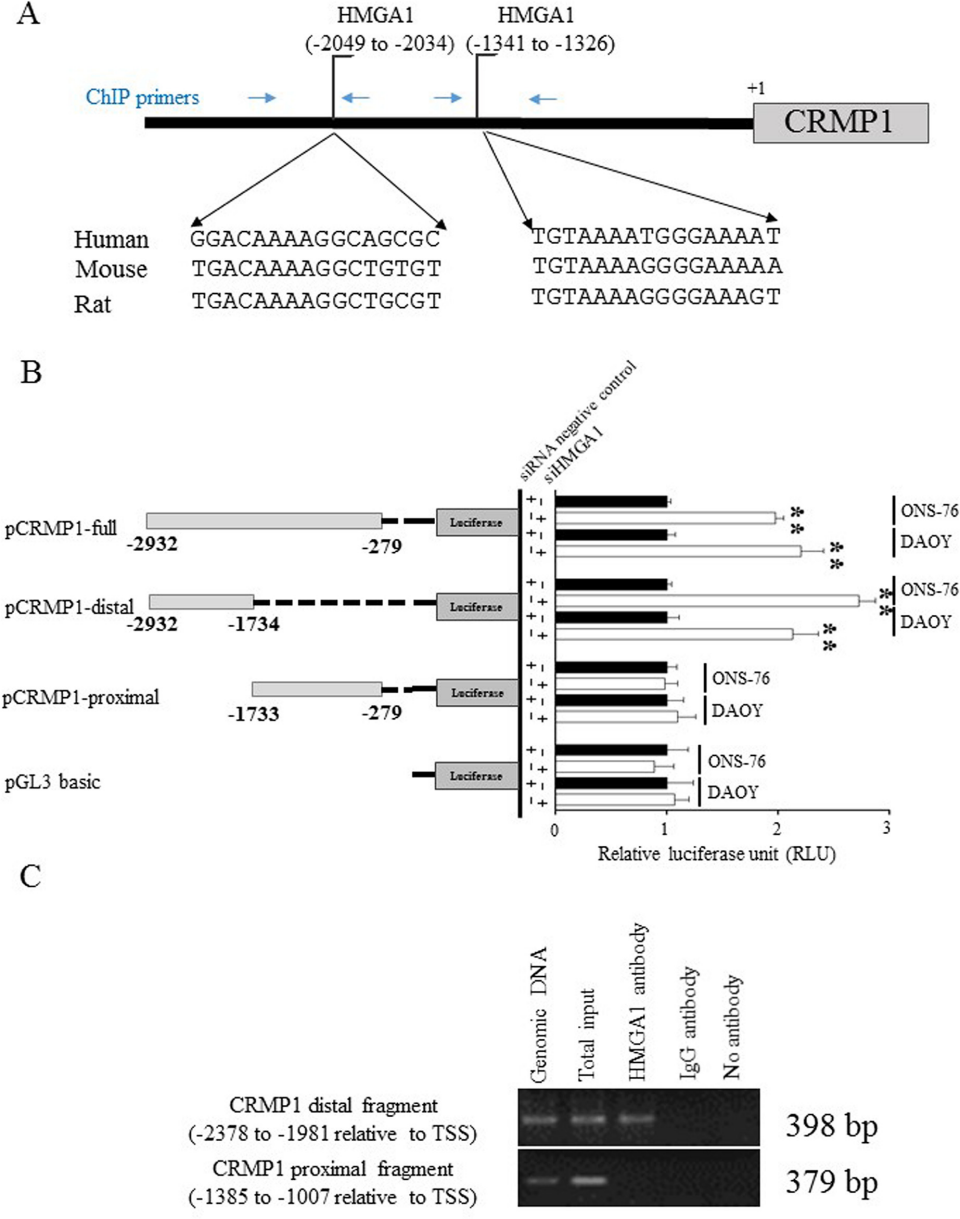
CRMP1 was regulated by HMGA1 through direct interaction on the CRMP1 promoter. (A) DNA sequence of upstream of CRMP1 promoter was analyzed by ConSite software. Two regions, nt -2049 to -2034 and nt -1341 to -1326 relative to transcription start site (indicated by the brackets), were identified as potential HGMA1 binding sites. The sequences at the bottom show the alignment of potential HMGA1 binding sites on the human, mouse, and rat CRMP1 genes using ClustalW2 Multiple Sequence Alignment. The blue arrows indicated locations of ChIP primers. (B) HMGA1 regulated CRMP1 transcriptional activity. DAOY and ONS-76 cells were transiently co-transfected with different CRMP1-luciferase vectors, pRL-CMV renilla plasmid, and HMGA1-specific siRNA or control siRNA. HMGA1 knockdown effect on CRMP1 promoter activity was examined by measuring the luciferase activity at 48h post-transfection. Depletion of HMGA1 in cells treated with pCRMP1-full or pCRMP1-distal resulted in a significant increase in luciferase activity (** indicates p<0.01). The data represent mean±SD of three independent triplicate experiments. (C) Chromatin immunoprecipitation (ChIP) analysis of HMGA1 binding to CRMP1 gene promoter. Sheared cross-linked chromatin extracted from DAOY cells was immunoprecipated with HMGA1-specific (lane 3) or rabbit italic gamma-globulin antibody as negative control (lane 4) or no antibody as no antibody control (lane 5). The DNA freed from chromatin was amplified using two pairs of primers (primer positions are shown in Fig 4A). Genomic DNA (lane 1) and Input DNA (lane 2) were included as controls.

We next investigated the molecular mechanism by which HMGA1 regulated CRMP1 expression. HMGA1 is an architectural transcription factor that is characterized by AT-hook DNA binding domains, and is capable of binding to the narrow minor groove of DNA to regulate transcription activity and gene expression [31–34]. Using bioinformatics software ConSite [35], we identified two putative HMGA1 binding sites within 3-kb upstream of the CRMP1 transcriptional start site (TSS) determined by the published mRNA sequence in NCBI GenBank (accession no. NM_001313). One potential binding site was located at distal position -2049 through -2034 and the other was found at proximal position -1341 through -1326 relative to TSS (Fig 4A). Multiple Sequencing Alignments software ClustalW2 revealed a 71% sequence homologous to mouse and 81% homologous to rat in human distal binding sequence (Fig 4A). The proximal binding sequence showed 88% homologous to mouse and rat (Fig 4A). We hypothesized that HMGA1 regulates CRMP1 through binding to the CRMP1 promoter.

To demonstrate the regulation of HMGA1 on CRMP1 activity, we cloned different fragments of CRMP1 promoter into luciferase reporter constructs and generated three CRMP1-luciferase vectors, namely pCRMP1-full, and pCRMP1-distal, and pCRMP1-proximal (Fig 4B). Construction of CRMP1-luciferase vectors was done using primers listed in S2 Table. These CRMP1-luciferase constructs were tested for promoter activity in condition which HMGA1 was depleted by gene-specific siRNA in two human MB cell lines, DAOY and ONS-76. The efficacy of siRNA against HMGA1 was analyzed by quantitative RT-PCR and western blot (S2 Fig). We observed luciferase activity was significant increased by 2.2-fold in DAOY and 2-fold in ONS-76 when cells were cotransfected with CRMP1-full plasmid covered nts -2932 to -279 of CRMP1 and a siRNA against HMGA1 (p<0.01; Fig 4B). DAOY and ONS-76 cells transfected with pCRMP1-distal containing nts -2932 to -1734 of CRMP1 also exhibited an elevation in luciferase activity by 2.1- and 2.7-fold respectively (p<0.01). However, enhance in promoter activity was not detected when cells were introduced with pCRMP1-proximal construct bearing nts -1733 to -279 of CRMP1 gene. The results indicated that important DNA sequence for HMGA1 regulation resided within the distal region of CRMP1 promoter.

### HMGA1 interacted with CRMP1 promoter

To provide evidence for a direct binding of HMGA1 to the promoter of CRMP1 *in vivo*, we performed chromatin immunoprecipitations (ChIP) using an antibody against HMGA1. We cross-linked protein–DNA interactions in DAOY cells that expressed high endogenous level of HMGA1 [28]. We then used PCR to assay a 398-bp fragment located on the distal region of the CRMP1 gene (-2378 to -1981 relative to the TSS), and a 379-bp fragment located on the proximal region of the gene (-1385 to -1007). The results revealed a strong binding of HMGA1 to distal region of CRMP1 promoter (Fig 4C). However, we did not detect binding of HMGA1 to the proximal region of CRMP1 promoter (Fig 4C). And, PCR amplification was not found in the IgG control (Fig 4C). The results indicated that HMGA1 interacted a 398-bp fragment containing putative CRMP1 binding site on the distal region of CRMP1 promoter and the relevance of the proximal region might be less under the biological conditions studied.

### Characterization of CRMP1 stably expressed cell lines

**Fig 5:**
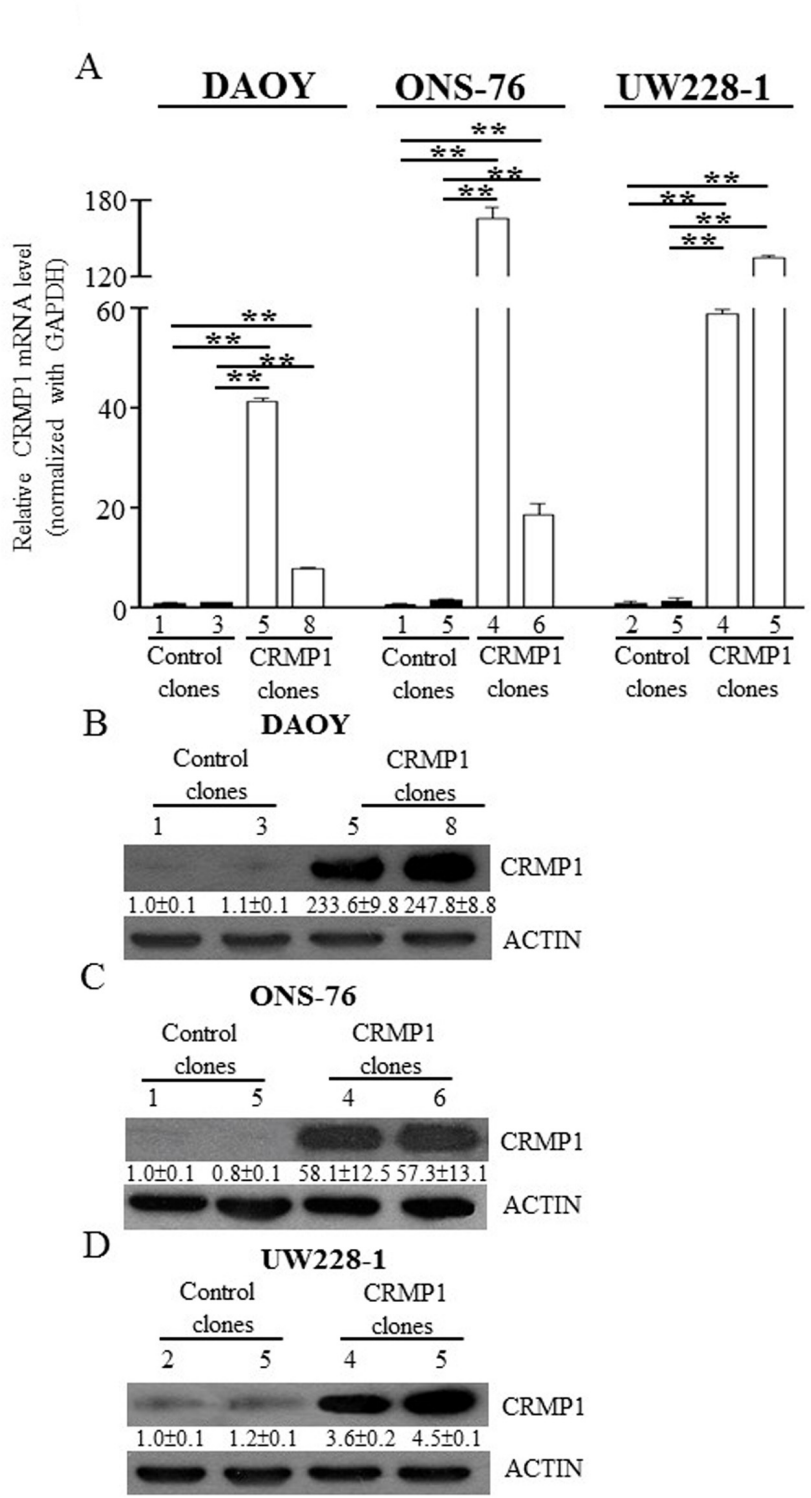
Generation of CRMP1 stably expressed MB cells. DAOY, ONS-76, and UW228-1 cells were transfected with pcDNA3.1-CRMP1 or pcDNA3.1 control plasmids. Stable clones were isolated by selection with G418. (A) Quantitative RT-PCR of CRMP1 in CRMP1 stably expressed cells and control cells. CRMP1 expression was normalized with GAPDH. The columns are expressed as mean±SD (** indicates p<0.01). (B) Western blot analysis of CRMP1 in clones of DAOY (top), ONS-76 (middle), and UW228-1 (bottom). The numbers under the protein bands indicated the relative band intensity normalized with ACTIN, and the control clones were set to 1. ImageJ software was used for quantification.

To elucidate the roles of CRMP1 in MB biology, we established stably expressing CRMP1 clones. The human MB cell lines DAOY, ONS-76, and UW228-1 were transfected with pcDNA3.1-CRMP1 containing the full length of cDNA of CRMP1 or control vector (pcDNA3.1). They were then selected in the presence of G418 and clones were allowed to expand. These three cell lines exhibited minimal expression level of CRMP1, with 28- to 1700-fold downregulation of CRMP1 compared with the mean of normal cerebella. Two CRMP1 expressing clones (named: cell line-CRMP1-#) and two control clones (named: cell line-control-#) of each cell lines were selected. Quantitative RT-PCR and western blot analysis validated the upregulation of CRMP1 in these MB clones. As shown in Fig 5A, expression of CRMP1 was sharply enhanced in DAOY-CRMP1-#5 and #8 compared with DAOY-control-#1 and #3 (p<0.01). Western blot analysis revealed that the protein level of CRMP1 was increased by 233.6-fold in DAOY-CRMP1-#5 and 247.8-fold in DAOY-CRMP1-#8 (Fig 5B). Similar and statistically significant upregulation of CRMP1 was observed in ONS-76 and UW228-1 clones stably expressing CRMP1 as shown in Fig 5A, 5C and 5D (p<0.01).

### CRMP1 inhibited cell proliferation in vitro

**Fig 6:**
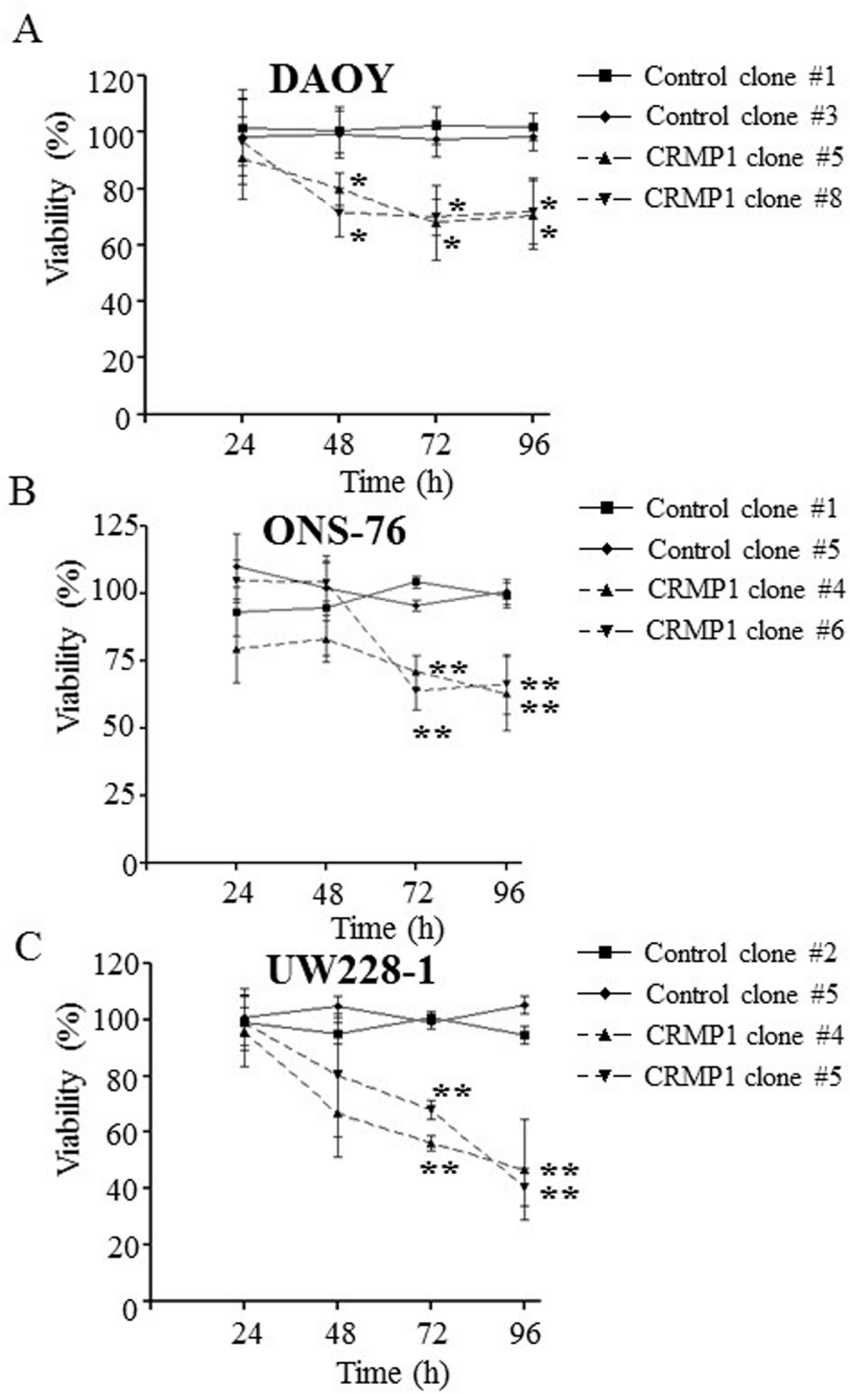
Expression of CRMP1 induced suppression of cell proliferation in MB cells. Clones of (A) DAOY, (B) ONS-76, and (C) UW228-1 were assessed for cell proliferation by MTT assay every 24h for a total of four days. Quadruplicates were performed in each experiment. The data represent the result of three individual experiments. * indicates p<0.05; ** indicates p<0.01.

To investigate the roles of CRMP1 in MB biology, we first measured cell proliferation of CRMP1- and control- clones. MB cells were plated on culture plates and allowed to grow. MTT assay was then performed every 24h for a consecutive four days. Compared to the control clones, DAOY-CRMP1-#5 and-#8 showed significant decrease in cell proliferation as early as 48 h after seeding (Fig 6A; p<0.05). Cell viability was suppressed by 19–21% in DAOY-CRMP1-#5 and by 28–29% in DAOY-CRMP1-#8 compared with the two control clones. Prolonged cell incubation further reduced cell proliferation of DAOY-CRMP1 clones. To rule out the clonal difference and to confirm the suppression of cell proliferation induced by CRMP1, we evaluated cell proliferation in clones derived from ONS-76 and UW228-1 cells. Similarly, expression of CRMP1 resulted in marked suppression in cell proliferation in at 72h and 96h (Fig 6B and 6C; p<0.01). Taken together, our data demonstrated the involvement of CRMP1 in regulating MB cell growth.

### CRMP1 suppressed cell migration and invasion

**Fig 7:**
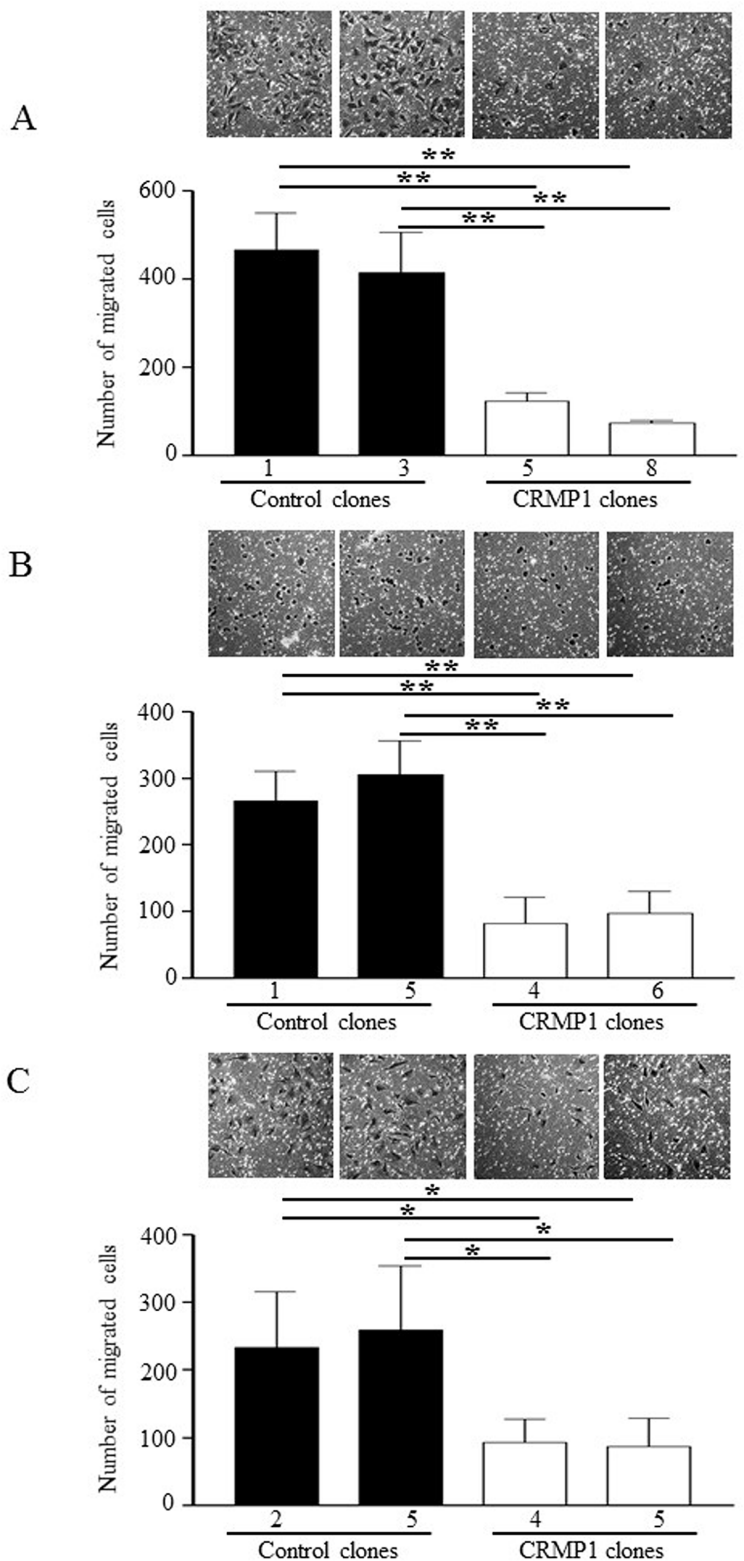
Expression of CRMP1 inhibited cell migration. Clones of (A) DAOY, (B) ONS-76, and (C) UW228-1 stably expressing CRMP1 were examined for migration by transwell assay. The bars shown as mean±SD of the number of cells migrated across uncoated transwell membranes in three independent experiments. Representative images are shown on top of bar graphs. * indicates p<0.05; ** indicates p<0.01.

**Fig 8:**
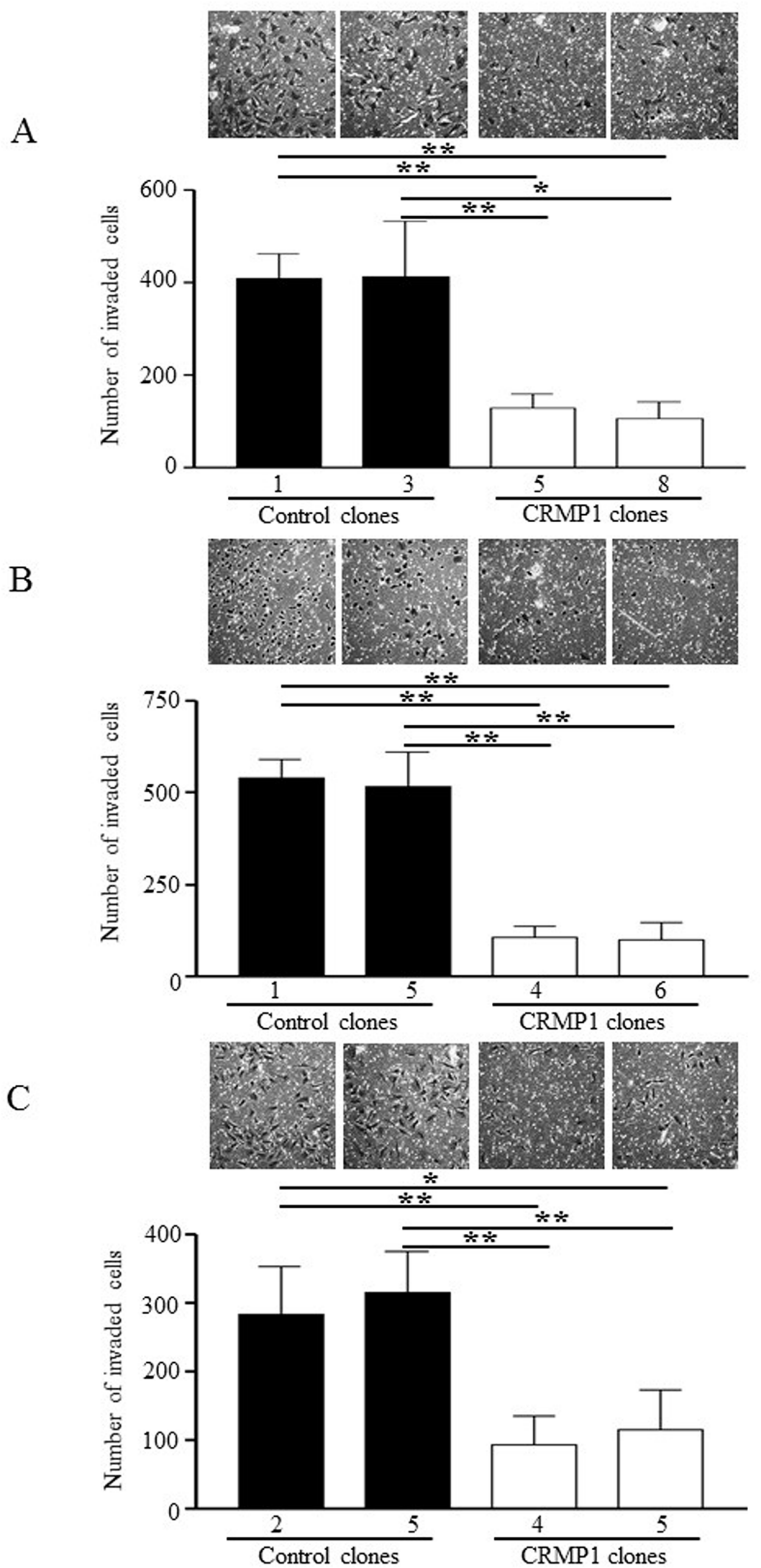
Effects of CRMP1 expression on invasion. Clones of (A) DAOY, (B) ONS-76, and (C) UW228-1 cells evaluated for invasion by matrigel invastion assay. Five random fields on each membrane were counted for the number of invaded cells. The bars illustrate mean±SD of the number of invaded cells in three separate experiments. On top of bar graphs shows representative photomicrographs. * indicates p<0.05; ** indicates p<0.01.

Next, we explored the consequences of CRMP1 expression on migration and invasion. Trans-well migration and invasion assays were conducted to measure migration and invasion capacity, respectively. As illustrated in Fig 7A–7C, expression of CRMP1 in DAOY-CRMP1-#5 strongly repressed the number of cells migrated through the microporous membrane by 70–74% compared with the two control cells (Fig 7A; p<0.01). The other clone DAOY-CRMP1-#8 exhibited reduction in migration potential by 82–84% (Fig 7A; p<0.01). The decrease in cell migration by CRMP1 was also validated in ONS-76 and UW228-1 clones stably expressing CRMP1 (Fig 7B and 7C; p<0.05). Invasion assay performed with pre-coated Matrigel membrane revealed that expression of CRMP1 resulted in strong impairment in invasion ability (Fig 8A–8C). The number of invaded cells was prominently decreased by 68–69% in DAOY-CRMP1#5 and by 74–75% in DAOY-CRMP1-#8 when compared with those of control cells (Fig 8A; p<0.05). ONS-76 and UW228-1 clones stably expressing CRMP1 also displayed significant diminish in invasion ability, further illustrating the roles of CRMP1 in suppression of cell invasion (Fig 8B and 8C; p<0.01).

### Overexpression CRMP1 impaired filopodia and intense stress fibers formation

**Fig 9:**
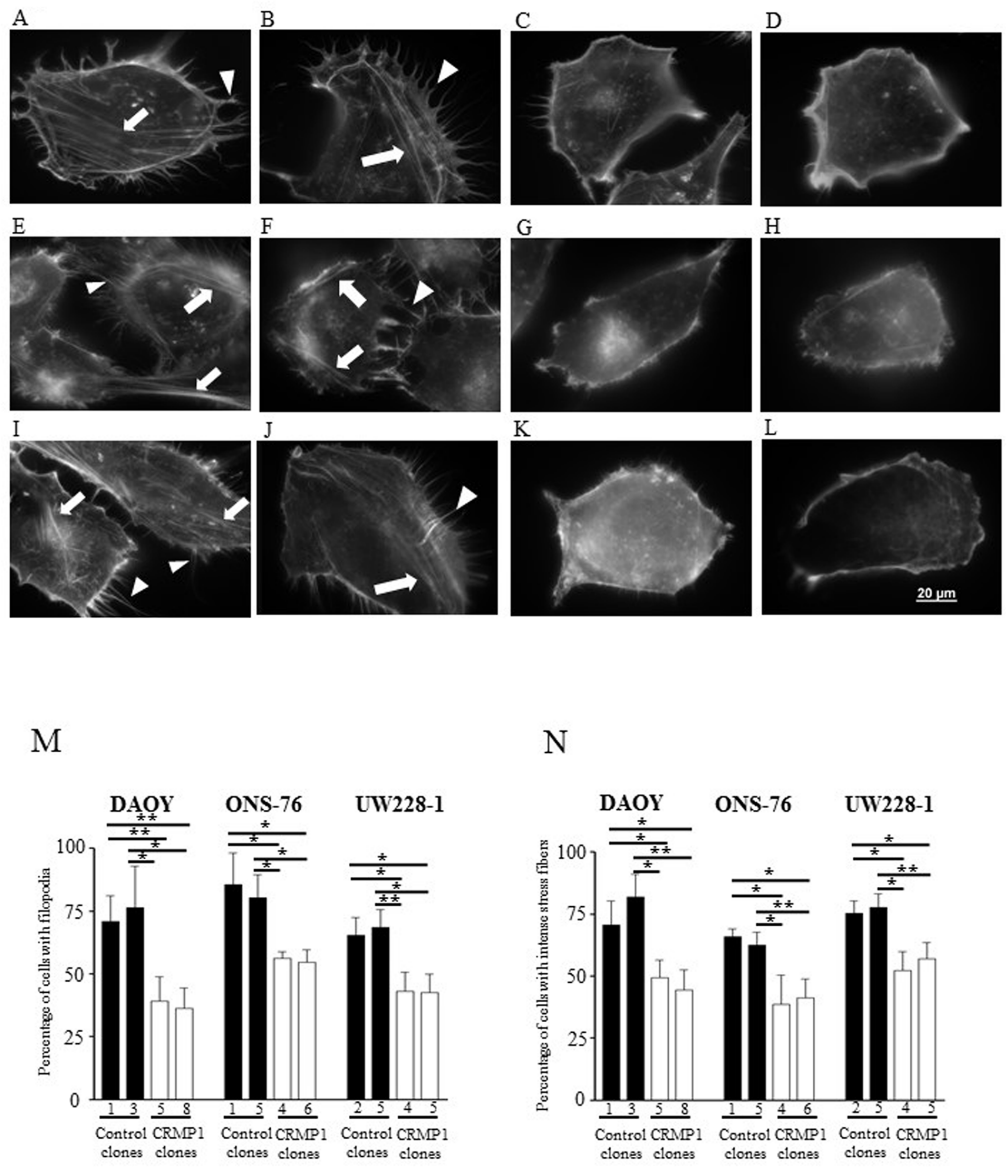
Overexpression of CRMP1 suppressed filopodia and intense stress fibers formation. F-actin organization was visualized using TRITC-conjugated phalloidin. Representative images of actin cytoskeleton structures in (A) DAOY-control-#1, (B) DAOY-control-#3, (C) DAOY-CRMP1-#5, (D) DAOY-CRMP1-#8, (E) ONS-76-control#1, (F) ONS-76-control#5, (G) ONS-76-CRMP1#4, (H) ONS-76-CRMP1#6, (I) UW228-1-control-#2, (J) UW228-1-control-#5, (K) UW228-1-CRMP1-#4, and (L) UW228-1-CRMP1-#6. Arrowheads represent filopodia and arrows indicate intense stress fibers. (M) The percentage of cells with filopodial protrusions was decreased in MB clones stably expressing CRMP1. A minimum of 100 individual cells of each clone were counted, and cells showing more than 20 filopodia were considered positive. (N) A minimum of 100 individual cells of each clone were counted to determine the percentage of cells bearing stress fibers in cell body. Data show the mean±SD from three individual experiments.

Lastly, we analyzed the roles of CRMP1 in actin cytoskeleton structure formation. Palloidin staining were performed to determine the abundance of filopodia and stress fibers in stably expressing CRMP1 clones and control clones. The results revealed that increased CRMP1 in MB clones substantially decreased filopodia formation. The percentage of cells with filopodia protruding from cell surface was decreased from 71.0%±10.2% in DAOY-control-#1 and 76.5%±16.6% in DAOY-control-#3 to 39.2%±9.6% in DAOY-CRMP1-#5 and 36.3%±8.2% in DAOY-CRMP1-#8 (Fig 9A–9D and M; p<0.05). The disruption in filopodia formation was also appeared in ONS-76 and UW228-1 clones stably expressing CRMP1 (Fig 9E–9M; p<0.05). Furthermore, we detected a marked reduction in intense stress fibers formation in MB cells expressing CRMP1. In DAOY cells, the percentage of intense stress fibers was dropped from 78.7–82.0% in two control clones to 44.6–50.3% in CRMP1 expressing clones (Fig 9A–9D and N; p<0.05). In addition, the well-organized stress fibers appeared in control clones were no longer detected in CRMP1 expressing clones. The same observations were made in ONS-76 and UW228-1 clones expressing CRMP1 when compared with their respective controls (Fig 9E–9L and N; p<0.05). The results suggested that CRMP1 modulated filopodia and stress fibers formation.

## Discussion

CRMP1 is a cytosolic protein that controls neurite formation by regulating cytoskeletal organization and modulates axon outgrowth via semaphorin signaling transduction pathway [18; 36–38]. Recent studies provide evidence that CRMP1 participates in tumor progression. In lung cancer, CRMP1 expression is significantly downregulated compared to adjacent normal tissue [39]. Moreover, transcript abundance of CRMP1 was negatively associated with tumor invasiveness [39]. In addition, patients exhibiting low CRMP1 mRNA level presented with more advanced disease stage [26]. In contrast, patients with high CRMP1 expression had a much longer survival [26]. In vitro study demonstrated that invasive activity was dramatically reduced in CRMP1 expressing lung cancer cells [26].

In our previous work, we demonstrated HMGA1 is aberrantly overexpressed in MB [28]. We further identified a list of potential HMGA1-regulated genes including CRMP1 by conducting a global gene expression microarray of HMGA1-depleted MB cells. We confirmed increased expression of CRMP1 in MB cells treated with siRNA against HMGA1. Overall, we speculated then that CRMP1 is negatively regulated by HMGA1 and it is functionally important in MB tumor biology.

In this study, for the first time, we showed that a 398-bp sequence residing between -2378 to -1981 on the promoter region of CRMP1 directly binds to HMGA1 in vivo by chromatin immunoprecipitation experiment. Moreover, this binding exerts a functional consequence-by luciferase assay, repression of HMGA1 induced a substantially increase in the activity of CRMP1 promoter. In clinical samples of MB, we found a significant negative correlation between CRMP1 and HMGA1. Our result was further confirmed by a public global gene expression dataset of 189 MB. We attempted to validate the finding in a second expression profiling dataset of 103 samples. However, we would only observe a trend towards negative correlation. We postulated that differences in array platform and probe design might explain such variance. Collectively, these data highly suggest that HMGA1 interacts with CRMP1 promoter to negatively regulate its expression.

In the present study, we also described downregulation of CRMP1 in 37.5% of MB and 100% of MB cell lines examined. Aberrant expression of CRMP1 has been reported in lung cancer [26]. Our results suggest that dysregulation of CRMP1 is a common event in MB, and upregulation of HMGA1 as shown in our previous study may be a mechanism contributed to the downregulation of CRMP1 in MB.

Although reduced CRMP1 expression was associated with clinical outcomes in lung cancer [26], we did not detect a link between CRMP1 and progression-free survival (PFS) or overall survival (OS) in either our panel of samples or the two expression dataset cohorts. Moreover, we identified statistically significant reduction of CRMP1 in Group 3 tumors when we analyzed the two independent expression profiling datasets. However, in our own sample cohort, we found no difference in CRMP1 among molecular subgroups, possibly due to the small sample size of individual subgroups.

Lastly, we established CRMP1 expressing cells to delineate the functional roles of CRMP1 in MB. To obtain reliable and reproducible results, we selected two representative stable clones from each of the three cell lines for functional studies. By MTT assay, we illustrated that enforced CRMP1 expression inhibited cell proliferation, an observation that was consistently found all different MB clones examined. This is the first report demonstrating a growth suppressive effect of CRMP1 in MB. We also observed that expression of CRMP1 decreased migration and invasion potential and resulted in fewer filopodia and intense stress fibers, suggesting that CRMP1 has multiple functions in MB biology. Our results are in concordance with previous finding demonstrating involvement of CRMP1 in cancer invasion in lung, gliomas and prolactin pituitary tumors [23–24; 26]. As the actin cytoskeleton is key part to migration and invasion, it is reasonable to postulate that the impairment of migration and invasion in CRMP1 expressing cells is due to defect in the formation of in filopodia and intense stress fiber [40–42].

In summary, we show that HMGA1 negatively regulates CRMP1 gene expression by binding to the CRMP1 promoter. CRMP1 acts to suppress tumor proliferation, migration, invasion and formation of filopodia and intense stress fibers in MB. This study shows that CRMP1 is an important player involved in the pathogenesis of MB.

## Supporting Information

S1 Table Clinicopathological information of 32 MB examined in this study. (TIF)

S2 Table Primer sequences for CRMP1 luciferase constructs used in this study. (TIF)

S1 Fig Downregulation of CRMP1 transcript abundance in Group 3 tumors. Data were analyzed from (A) Cho et al. study and (B) Northcott et al. study.
(TIF)

S2 Fig Downregulation of HMGA1 by siRNA. (A) Transcript and (B) protein abundance of HMGA1 in DAOY and ONS-76 cells after 48h transfection.
(TIF)
