# Supplementary figures and images for: CRMP1 Inhibits Proliferation of Medulloblastoma and Is Regulated by HMGA1
Source: PLoS One. 2015 May 26;10(5):e0127910. doi: 10.1371/journal.pone.0127910 (PMC4444180; doi:10.1371/journal.pone.0127910)

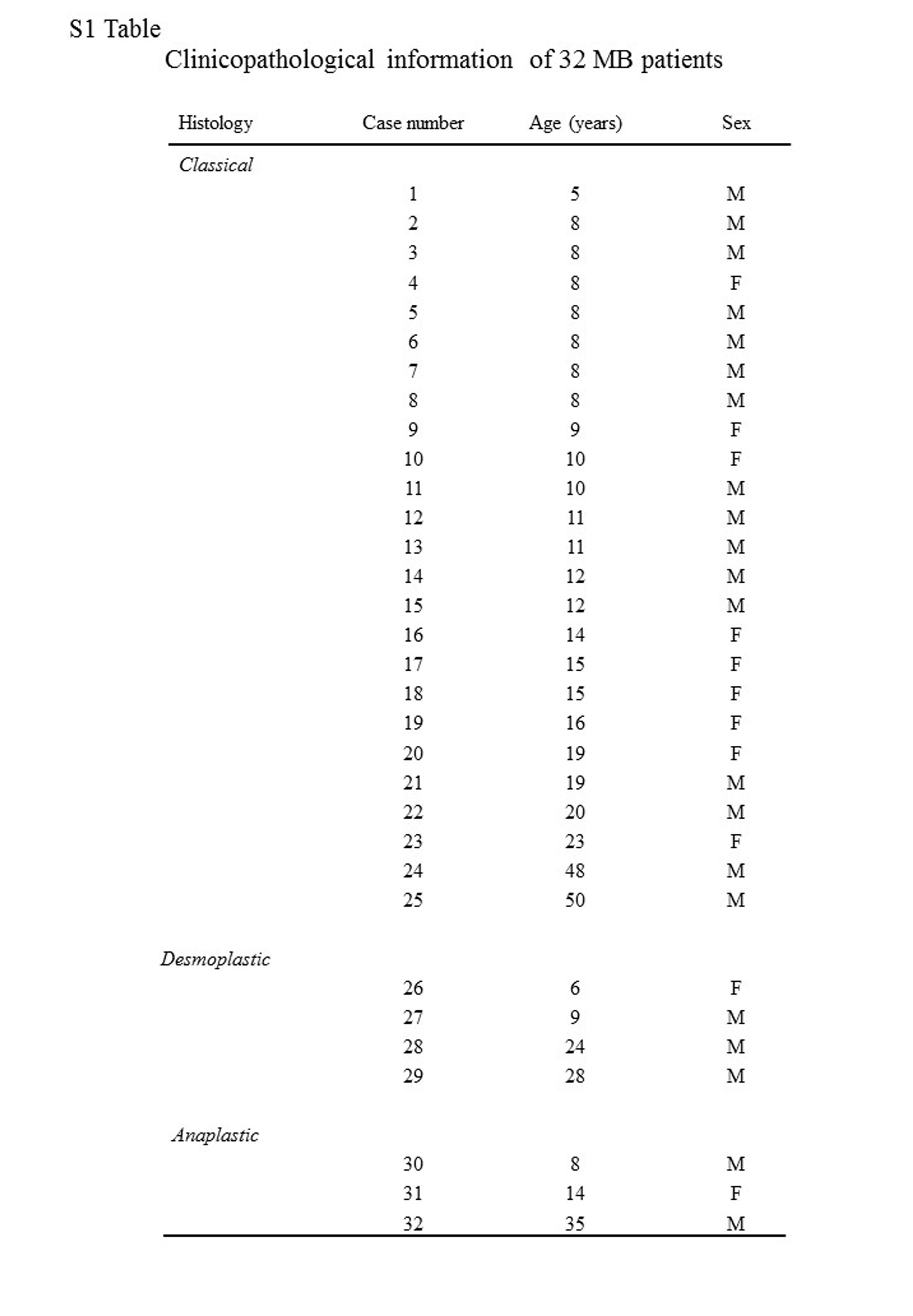

Supplement: S1 Table — (TIF) [file pone.0127910.s001.tif]

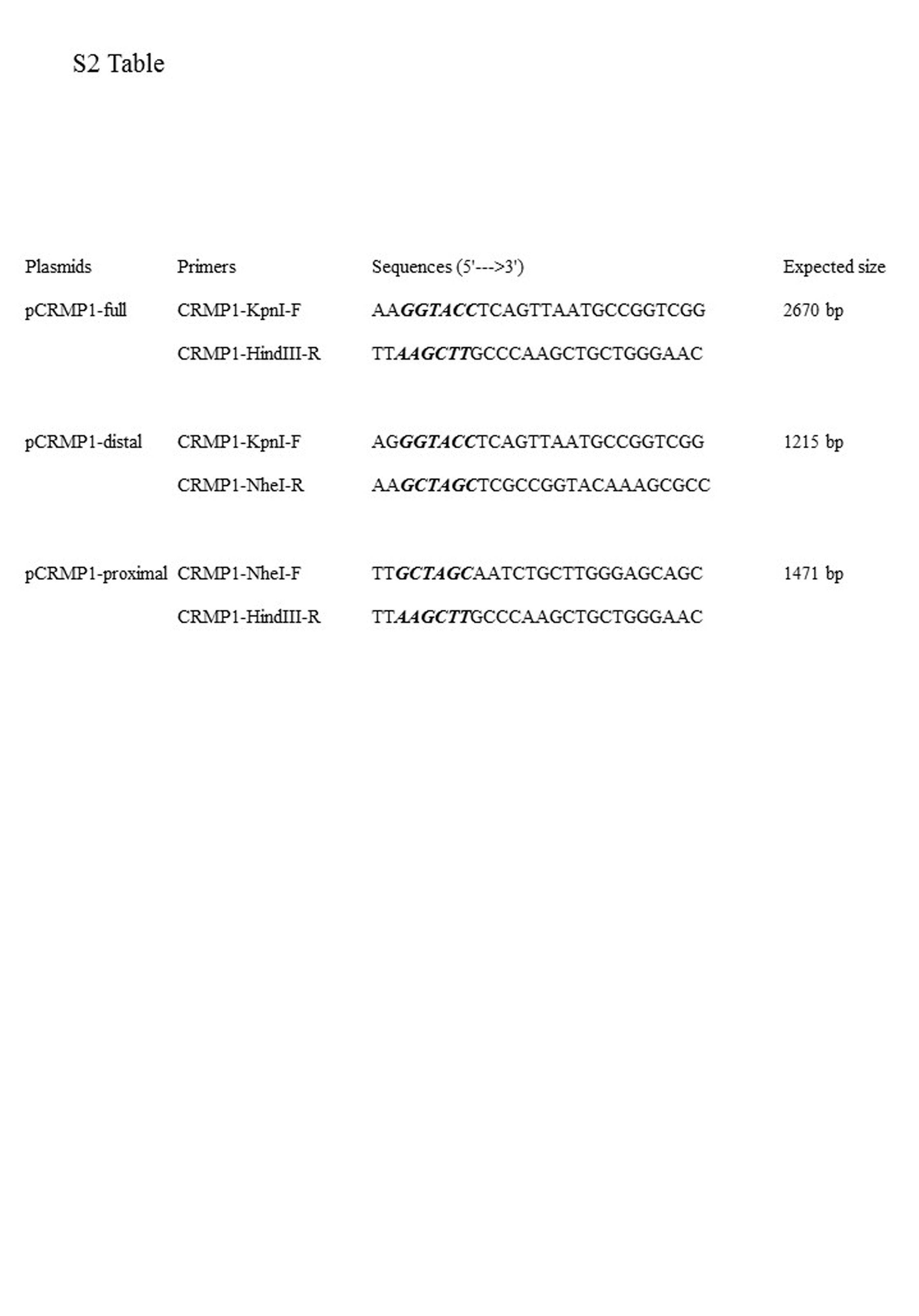

Supplement: S2 Table — (TIF) [file pone.0127910.s002.tif]

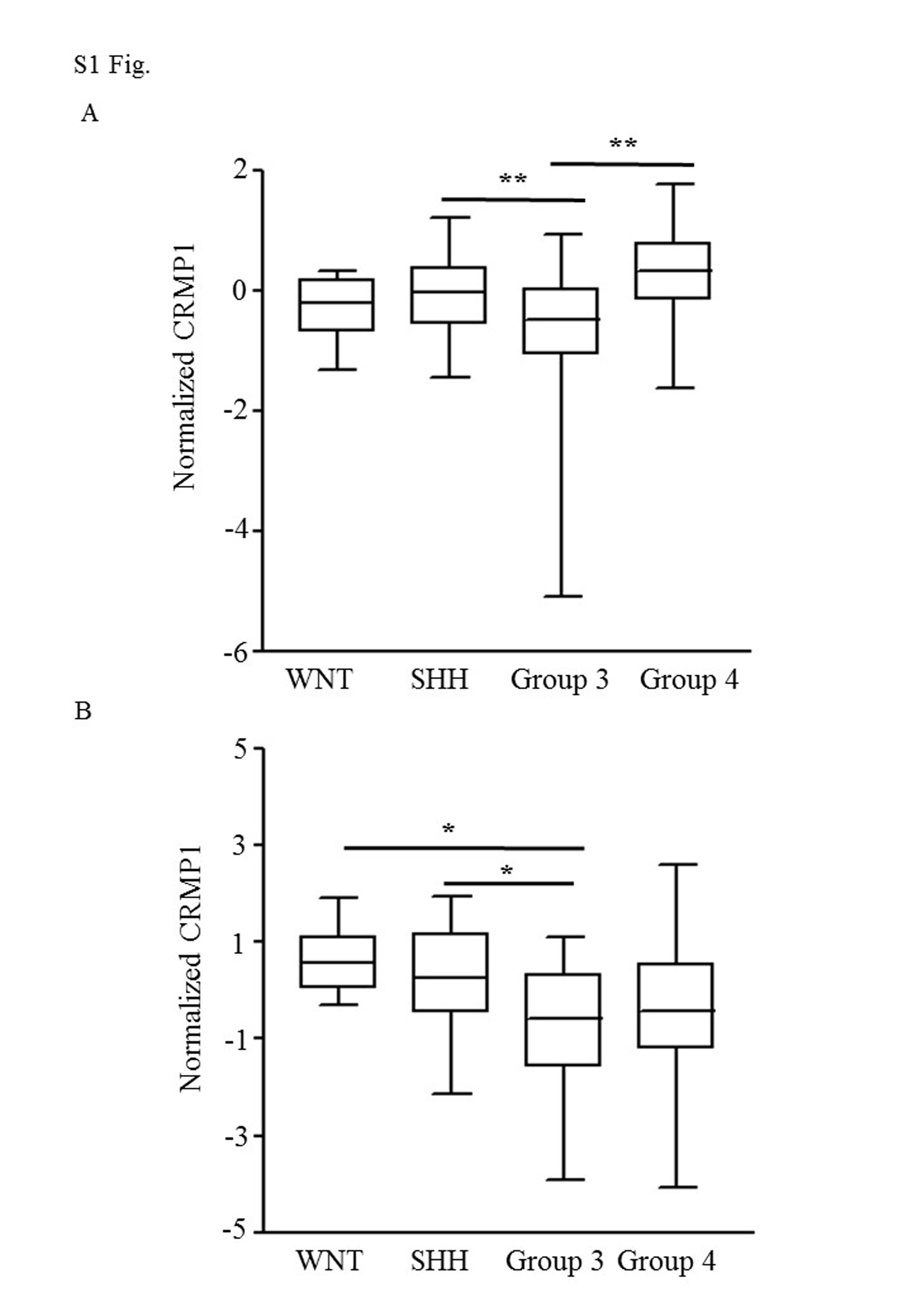

Supplement: S1 Fig — Data were analyzed from (A) Cho et al. study and (B) Northcott et al. study. (TIF) [file pone.0127910.s003.tif]

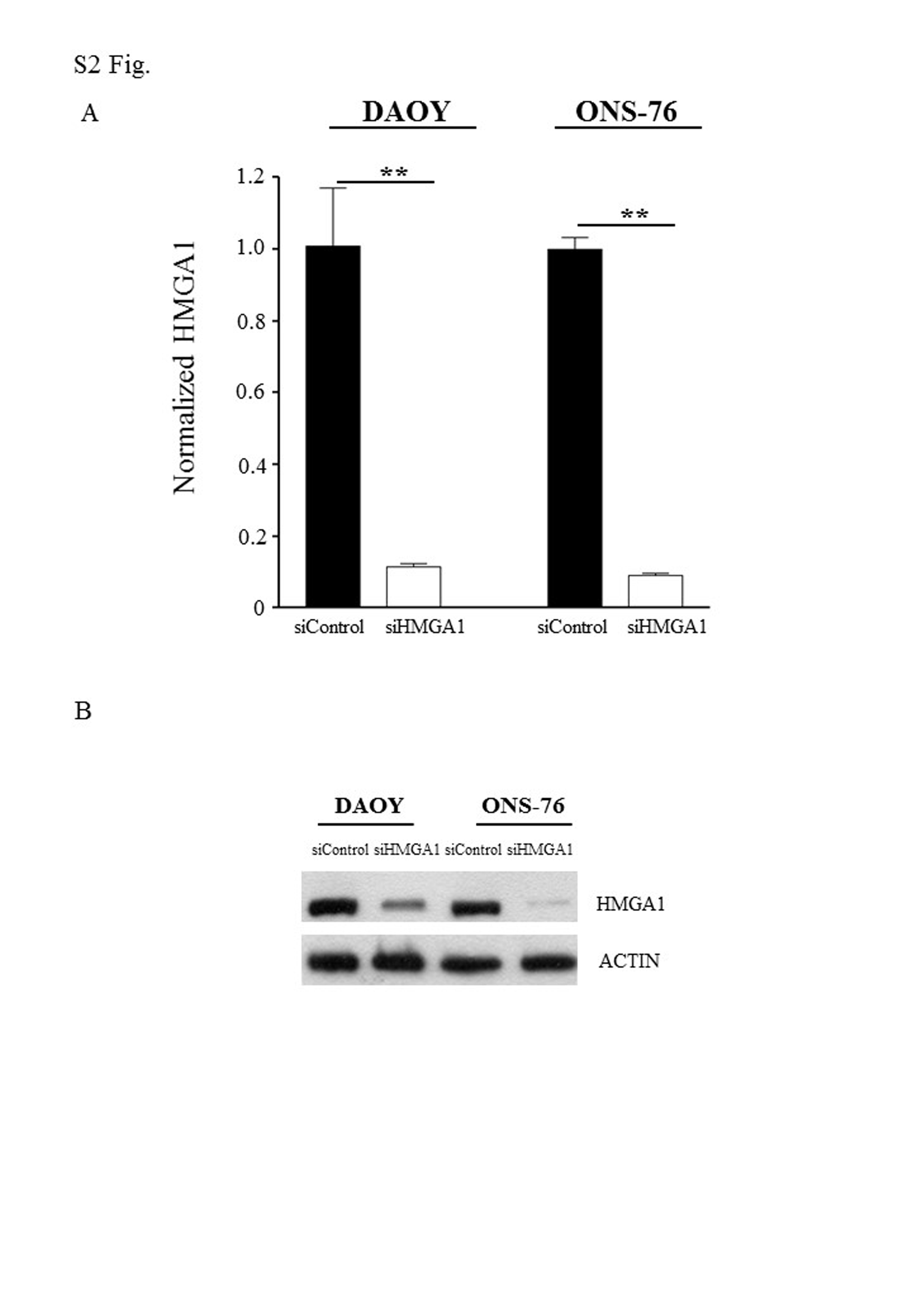

Supplement: S2 Fig — (A) Transcript and (B) protein abundance of HMGA1 in DAOY and ONS-76 cells after 48h transfection. (TIF) [file pone.0127910.s004.tif]
